# Automated Image Threshold Method Comparison for Conjunctival Vessel Quantification on Optical Coherence Tomography Angiography

**DOI:** 10.1167/tvst.11.7.15

**Published:** 2022-07-20

**Authors:** William W. Binotti, Daniel Saukkonen, Yashar Seyed-Razavi, Arsia Jamali, Pedram Hamrah

**Affiliations:** 1 Center for Translational Ocular Immunology, Tufts Medical Center, Tufts School of Medicine, Boston, MA, USA; 2 Cornea Department, New England Eye Center, Tufts Medical Center, Tufts School of Medicine, Boston, MA, USA

**Keywords:** OCTA, conjunctiva, vessel density, thresholding, binarization, conjunctival vessels, optical coherence tomography angiography

## Abstract

**Purpose:**

To determine the impact of image binarization and the best thresholding method for conjunctival optical coherence tomography angiography (OCTA).

**Methods:**

Vessel density (VD) of 14 OCTA conjunctival images (nine nasal and five temporal conjunctivas, and eight right and six left eyes) from normal subjects was analyzed. The binarization of gold-standard images, created by removing pixels that do not represent vessels on ImageJ software, was assessed by three masked graders to determine consistency of VD for images. Various thresholding methods on ImageJ, including manual, 1-, 2- and 3-step processes, were performed on unprocessed images for comparison. Interclass correlation coefficient (ICC) ≥0.750 were classified as good reliability and selected for calculation of the performance of the pixel location in the binarized images of each method.

**Results:**

Analysis of the gold-standard threshold method achieved an ICC of 0.816 with excellent agreement (*R*^2^ = 0.965, *P* < 0.001). From a total 28 different methods and variations performed, only nine methods performed with good reliability, including two 1-step thresholds, six 2-step thresholds, and one 3-step threshold method. Overall, 2-step threshold methods were more reliable than 3-step threshold methods. The 2-step method of Bandpass filter + Phansalkar local threshold (LT) showed the best performance with mean pixel accuracy of 86.9% ± 6.8%, area under the curve of 0.826, sensitivity of 79.0%, and specificity 86.1%.

**Conclusions:**

Bandpass filter + Phansalkar LT was the best method for VD measurement in conjunctival OCTA. Most commonly reported threshold methods showed unsatisfactory agreement. There is a need in the OCTA field for a standardized method to allow comparison between different studies.

**Translational Relevance:**

The proposed threshold method using a widely accessible and commonly used software provides an accurate VD measurement for future OCTA studies.

## Introduction

The eye receives a high volume of blood flow, where many microangiopathic pathologies can manifest. Quantifying capillaries and vessels in ocular tissue has been an important biomarker to identify healthy from diseased eyes.^1^^–^^4^ Optical coherence tomography angiography (OCTA) has become a mainstay in research and is growing for clinical practice and clinical trials.^5^^–^^9^ This technology allows noninvasive flow detection of blood vessels and is able to generate volumetric angiograms at micrometer resolution.^10^^,^^11^ In recent years, OCTA has expanded to the anterior segment and has been shown to be useful in a variety of diseases, such as corneal neovascularization, limbal stem cell deficiency, ocular tumors, and pterygium.^12^^–^^20^ The bulbar conjunctival vessels have been the focus of many studies for in vivo and noninvasive assessment of vascular parameters, because of its ease of access and capacity of reflecting ocular and systemic diseases.^21^^–^^24^

Despite the advances in vessel imaging technology, there are a wide range of image thresholding methods for vessel measurement without a consistent and standardized method for processing OCTA images and quantifying vessel parameters. Image thresholding or binarization is the process that converts a gray value image into pixels above a determined threshold value in “white” (1) and below the value in “black” (0). There are many methods in determining the threshold value of the image, for example a global threshold applies a single value over the entire image.^25^^,^^26^ Another method is using a local or adaptive threshold that applies different values according to regional variations of the image.^26^^,^^27^ Also, various image processing techniques have been used to remove background noise and improve image contrast, whether it is smoothing the image or applying different filters to remove small particles and noise.^25^^,^^28^ Moreover, many authors propose a combination of the different methods for thresholding.^25^^,^^28^

The most commonly used vessel parameter in OCTA is vessel density (VD), which quantifies the percentage of pixels that represent blood flow compared to the total pixels in the entire image. Consistency and reproducibility of these measurements are important for research studies. However, the method of thresholding has been shown to significantly alter their measurements in retinal and choroidal OCTA.^29^^,^^30^ Similar to the posterior segment, the anterior segment lacks a standardized threshold method for quantitative OCTA assessment. With increasing studies using OCTA for analysis, there is a need to better understand the impact of different threshold methods on vessel measurements and to establish a consistent and accurate method for binarization of vessels in OCTA images.^29^^,^^30^ We hypothesize that different threshold methods will affect the VD measurements in anterior segment OCTA. Accordingly, the aim of this study was to assess the agreement and accuracy of different threshold methods in quantifying VD on OCTA images of the conjunctiva, by comparing to their reference gold standard images.

## Methods

Images from healthy volunteers were selected from the OCT database at the Cornea Department of New England Eye Center, Department of Ophthalmology, Tufts Medical Center, Boston, MA. Subjects were retrospectively reviewed, and images were de-identified following the tenets of the Declaration of Helsinki and the study was approved by Tufts’ Institutional Review Board/Ethics committee (IRB no. 12530). Inclusion criteria was subjects with conjunctival images who performed the OCTA protocol adapted for anterior segment in the OCT database. Exclusion criteria were any ocular diseases, including dry eye disease, systemic diseases (i.e., diabetes, hypertension), ocular infection, history of recent ocular surgery, and images of poor quality with excessive noise and low clarity that would impede conjunctival vessel visualization. In contrast to previous methods established on a single OCTA image (generally the best image example), multiple images were used for threshold comparison to better represent the variability of gray value that occurs between OCTA images.

### OCTA Imaging Technique

The OCTA images were acquired by the spectral domain OCTA system, Avanti XR AngioVue (Optovue Inc., Freemont, CA, USA; version 2018.0.0.10). The 6 × 6 mm HD Retina scan mode was used with the Optovue anterior segment lens (Long Cornea Adaptive Module) and manual adjustments of Z motor, P motor, and focus to image the conjunctival surface. The system acquires volumetric scans of 400 × 400 A-scans at 70,000 A-scans per second, using a light source centered on 840 nm and a bandwidth of 45 nm. Flow is detected through motion contrast of repeated A-scans at the same location, and motion artifact was removed by 3D orthogonal registration and merging of two scans using the SSADA algorithm, thus producing a 5 µm axial resolution volumetric angiogram with the motion of the red blood cells within vessels.

Each image was exported in their 400 × 400 pixel format, analyzed and processed on open source software FIJI from ImageJ (National Institutes of Health, Bethesda, MD, USA). Because OCTA is a novel and adapted technology from the retinal to the anterior segment, images of the conjunctiva can present with variable background noise as compared to retinal images. To focus the analysis on the conjunctiva, the eyelids, eyelashes and cornea were carefully removed from all OCTA images before thresholding.

### Establishing Gold Standard Images

**Figure 1.**
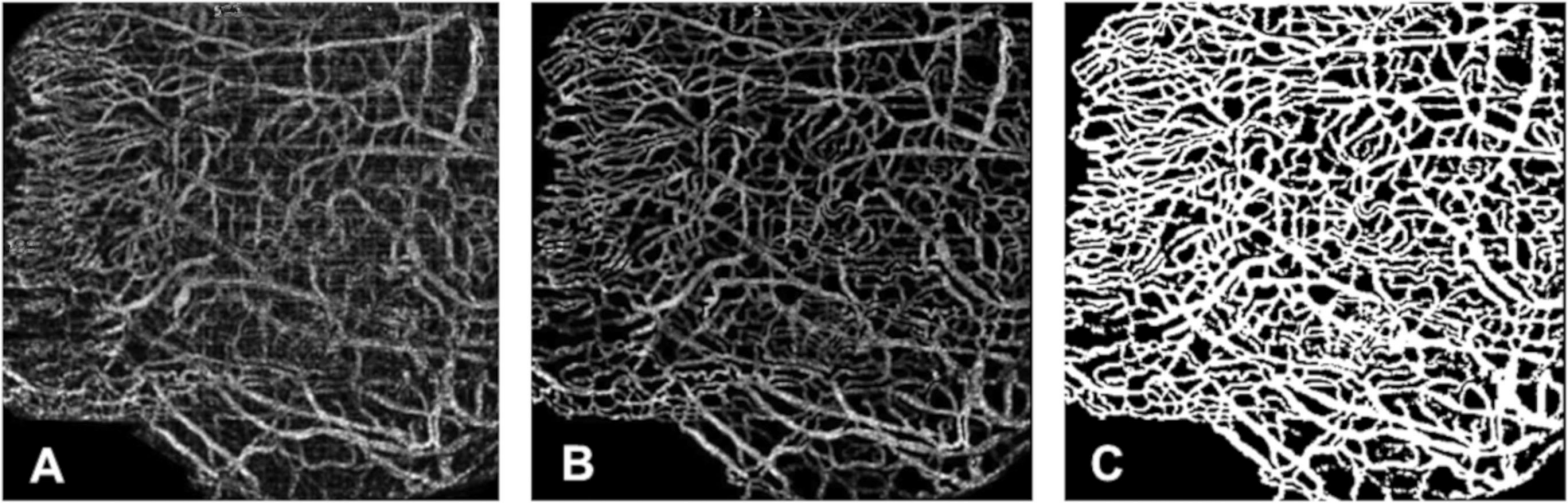
(**A**) OCTA RAW image of the nasal conjunctiva. (**B**) Processed image removing background noise and pixels that do not represent vessels. (**C**) Manually thresholded OCTA binary image.

Pixels that represented noise or imaging artifacts and clearly not a vascular component were manually removed in the selected images by an experienced OCTA grader (Figs. 1A, 1B). These images were then used as reference for subsequent thresholding processes. Next, the manual threshold was determined by three blinded and independent graders (W.B., D.S., Y.S.R.) with the aim of matching their respective reference image (Fig. 1C). After binarization (thresholding), the number of foreground pixels representing blood flow divided by the total pixels were represented as VD (% area). Each image generated from the mean threshold value of the three graders and its VD was considered the gold standard for the methodology comparison.

### Thresholding Methods

The following thresholding methods to acquire binary images for vessel quantification were selected based on previously published methods and applied to the unprocessed RAW images. The available methods consist of a variation of algorithms that transform a gray-value image into a binary image through a 1-step threshold, or a combination of image pre-processing and filters before the threshold (2-step or 3-step threshold methods). The additional steps before thresholding seek to reduce background noise, to remove pixels that do not represent vessels, or to create continuity of neighboring vessel pixels. Each VD from the thresholded images were compared to their respective gold standard image VD.

### 1-Step Threshold Methods

#### Manual Threshold

The same three blinded graders independently set the threshold on the RAW images according to their interpretation of the best representation of the vessels. Their threshold was applied to the entire (global) image. Their averaged VD for each image was used to compare to the gold standard.

#### Global Threshold

Global threshold applies a single threshold value to all pixels (global) within the entire image into either foreground pixels (1) or background pixels (0). The threshold value can be achieved by many methods (e.g., mean gray value of the image or Otsu algorithm), which compares the gray value variance of each pixel from the average gray value to determine the optimal cutoff value for the entire image (global) by minimizing the intraclass intensity variance.^26^

#### Local Threshold

Local threshold applies different local thresholds based on the neighboring gray values of each individual pixel, accounting for focal high-variability gray value in the image. Also known as an adaptive threshold method. Algorithms may use the mean or median gray values, Otsu (as previously described) or Phansalkar, which applies different local thresholds based on a combination of mean, standard deviation, and normalization of gray value within a 15-pixel radius. The latter is a method particularly used in low contrast images.^27^

### 2-Step Threshold Methods

**Figure 2.**
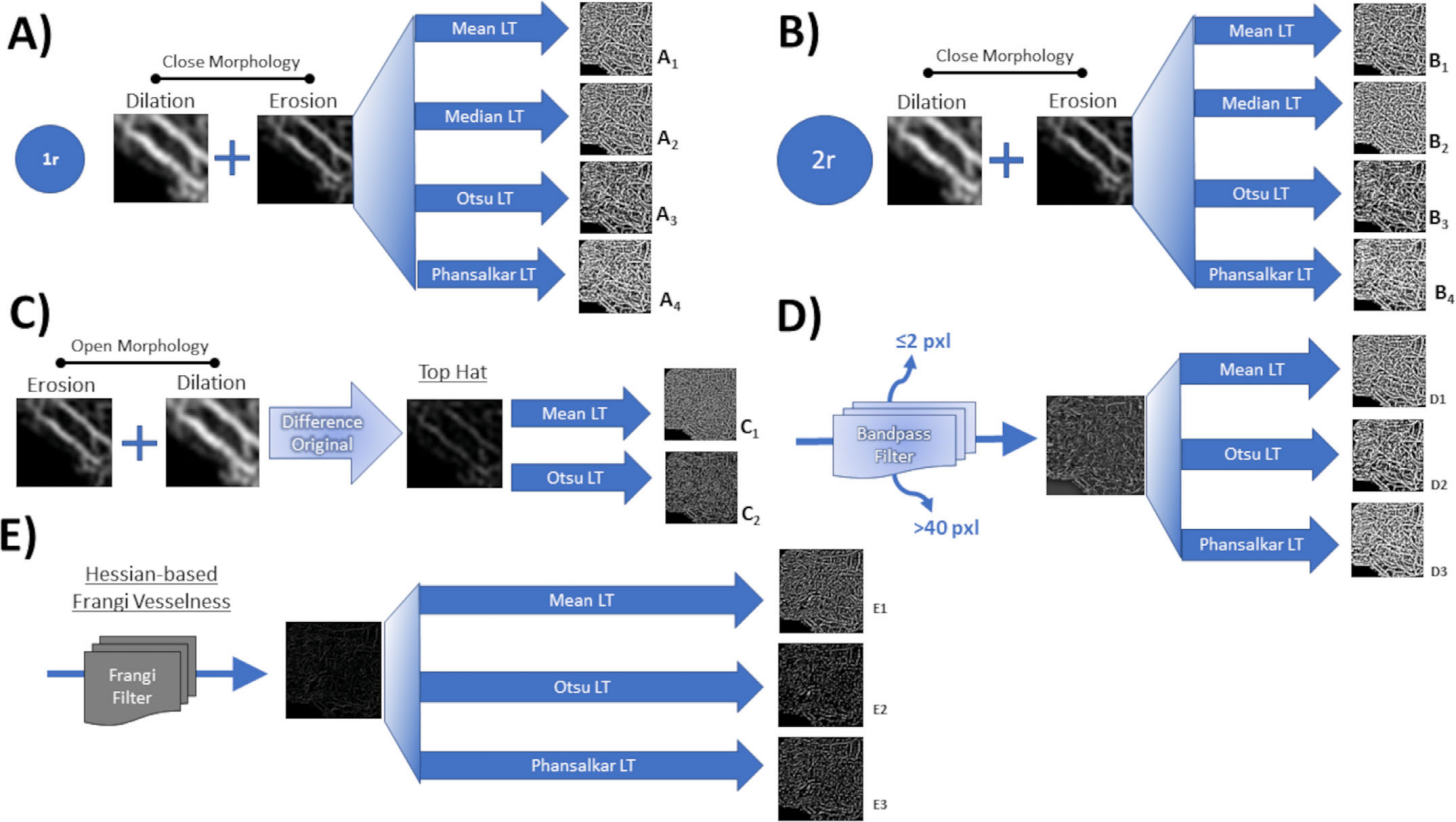
Diagram illustrating the different combined threshold methods. (A_1–4_) A closing morphology filter of a 1-pixel radius was applied; then, 4 different local threshold methods were analyzed, mean, median, Otsu and Phansalkar. (B_1–4_) A closing morphology filter of a two-pixel radius was applied; then, four different local threshold methods were analyzed, mean, median, Otsu, and Phansalkar. (C_1-2_) Top hat filter followed by a mean or an Otsu local threshold was applied. (D_1–3_) A bandpass filter that removes pixels ≤2 pixels and >40 pixels followed by a mean or Otsu or Phansalkar threshold was applied. (E_1–3_) A Frangi filter to identify vessel-like structures was applied, followed by mean or Otsu or Phansalkar local threshold.

**Figure 3.**
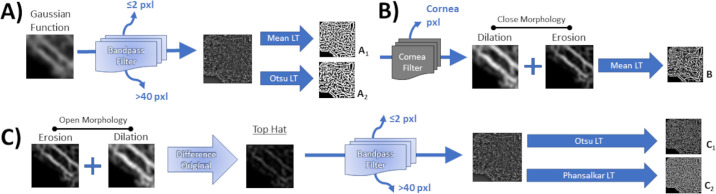
Diagram illustrating the different combined threshold methods. (A_1-2_) Gaussian smoothing filter with a sigma radius of four pixels was applied; then, a bandpass filter that removes pixels ≤2 pixels and >40 pixels followed by a mean or an Otsu local threshold was applied. (B) The average gray value of three corneal measurements was used to threshold image; then a close morphology followed by a mean threshold was applied. (C_1-2_) A top hat filter, then a bandpass filter followed an Otsu or a Phansalkar threshold was applied.

With the goal of creating threshold images that best represent the structure of interest (vessels in this case), many advocate a combination of filters or processing methods to enhance the structure of interest and minimize noise.^25^^,^^28^^,^^31^^–^^33^ Therefore a selection of commonly used combined methods were performed and are schematically represented in Figures 2 and 3.

#### Close Morphology With a One- or Two-Pixel Radius ± Local Threshold

First, by applying a closing morphology (image dilation followed by erosion), the algorithm “fills” the spaces between noncontiguous pixels within a one- or two-pixel radius of the neighboring pixels (removing “holes”). Then, four different local threshold methods were analyzed, mean, median, Otsu and Phansalkar (Fig. 2A_1-4_ and Fig. 2B_1-4_, respectively), based on previous descriptions.^25^^,^^28^^,^^31^ Close morphology is typically used in vascular imaging to improve pixel connectivity of vessel structures on threshold. One-pixel and two-pixel radius algorithm were compared to assess whether the size of the radius improved the method.

#### Top Hat Filter ± Local Threshold

Top hat filter consists of performing a morphological opening (image erosion followed by dilation) and then subtracting from the original image. This process removes particles that are smaller (pixels) than the structuring element and darker (lower gray value) than their surroundings; intended to remove the image noise, correct nonuniform lighting, and improve background contrast. Last, a mean and an Otsu local threshold was applied (Fig. 2C_1-2_, respectively).^28^^,^^32^^,^^34^

#### Bandpass Filter ± Local Threshold

A bandpass filter removes structures ≤2 pixels and ≥50 pixels, which removes the image noise and improved background contrast. No morphological or top hat processing was applied. Then, a local threshold method was applied, mean, Otsu, and Phansalkar (Fig. 2D_1-3_, respectively), as previously described. This combination was included to compare the effect of not performing the commonly used top hat filter for image analysis.^23^^,^^32^

#### Frangi Vesselness Filter ± Local Threshold

Hessian-based Frangi vesselness filter is an algorithm that is designed to enhance visualization of vessels on images by identifying multiscale pixels with vessel- or tube-like characteristics and enhancing vessel contrast and suppressing background noise. This method is commonly used in ultrasound, computerized tomography, magnetic resonance, and retinal photography vessel imaging.^35^^,^^36^ Last, local thresholds was applied as previously described (Fig. 2E_1-3_).

### 3-Step Threshold Methods

#### Bandpass Filter ± Gaussian Blur ± Local Threshold

First, the image is processed through a Gaussian blur (aka smoothing) with a sigma radius of four pixels, which is used to reduce overall image noise.^37^ Then, a Bandpass filter is applied that removes background pixels. Last, a mean and an Otsu local threshold was applied (Fig. 3A_1-2_, respectively).^17^^,^^34^^,^^38^

#### Cornea Global Filter ± Close Morphology ± Mean Local Threshold

Based on studies that use the foveal avascular zone to calculate the signal to noise ratio and determine the global threshold, the cornea (avascular in healthy subjects) was used for the global image filter.^16^^,^^32^^,^^39^ The average gray value of three measures of the cornea was used to filter pixels below that gray value. Subsequently, a close morphology was performed to fill “holes” within a 2-pixel radius, as previously described. Last, a mean local threshold was applied (Fig. 3B).^32^

#### Bandpass Filter ± Top Hat Filter ± local Threshold

A Bandpass filter was applied to remove small particles and background noise, then a top hat filter, as described previously. Last, an Ostu or Phansalkar local threshold was performed for binarization (Fig. 3C_1-2_).^23^^,^^32^^,^^39^

### Performance of the Methods

The final step of establishing the best threshold method is to determine not only the number of pixels thresholded as vessels in an image, i.e. vessel density (VD), but additionally the correct location of these pixels representing vessels, compared to the gold standard image. Therefore pixels of vascular flow (foreground pixels) that were present both on the image of interest and its gold standard were calculated as the true positives. Pixels that were included on the image of interest, but not on its gold standard, were calculated as false positives. Pixels that were included on the gold standard, but not on the image of interest, were calculated as false negatives. Pixels that were counted as non-vessels (background pixels) in both the image of interest and its gold standard were considered true negatives. Therefore the accuracy of the correct location of thresholded pixels were represented by adding the true-positive and the true-negative values (%) of each image then averaging their accuracy.

### Statistical Analysis

The Statistical Package for the Social Sciences software (ver. 17; SPSS Inc., Chicago, IL, USA) was used to analyze the data. Shapiro-Wilk test was performed to determine if the data was normally distributed. For the gold standard validation, the average between grader 2 and 3 were compared to grader 1 and their difference used to construct Bland-Altman plots and ICC test was performed to demonstrate the variability of the graders’ measures. Then, a linear regression was calculated and *R*^2^ for goodness-of-fit determined to demonstrate the association of the VD calculated from the new gold standard images with the VD calculated from manually-removed pixel images. Shapiro-Wilk normality test was performed on the mean standardized residuals to determine bias of the linear regression model. Bias was considered if *P* < 0.05.

Similarly, for the manual thresholding on RAW images, the average between graders 2 and 3 were compared to grader and the average of all 3 graders were compared to the gold standard VD. Furthermore, the VDs of each method were compared to the gold standard through ICCs with their 95% confidence intervals (lower bound − upper bound) and analyzed for absolute agreement. Only methods with good reliability, established as ICC ≥ 0.750,^40^ were considered for the pixel-location accuracy assessment. Mean accuracy was calculated by adding true positive and true negative values of all images represented with their 95% confidence intervals. Sensitivity was the calculated by dividing true positive by the sum of true positives and false negatives. Specificity was calculated by dividing the true negatives by the sum of true negatives and false positives. Area under the curve (AUC) was obtained by the average of sensitivity and specificity values.

## Results

**Figure 4.**
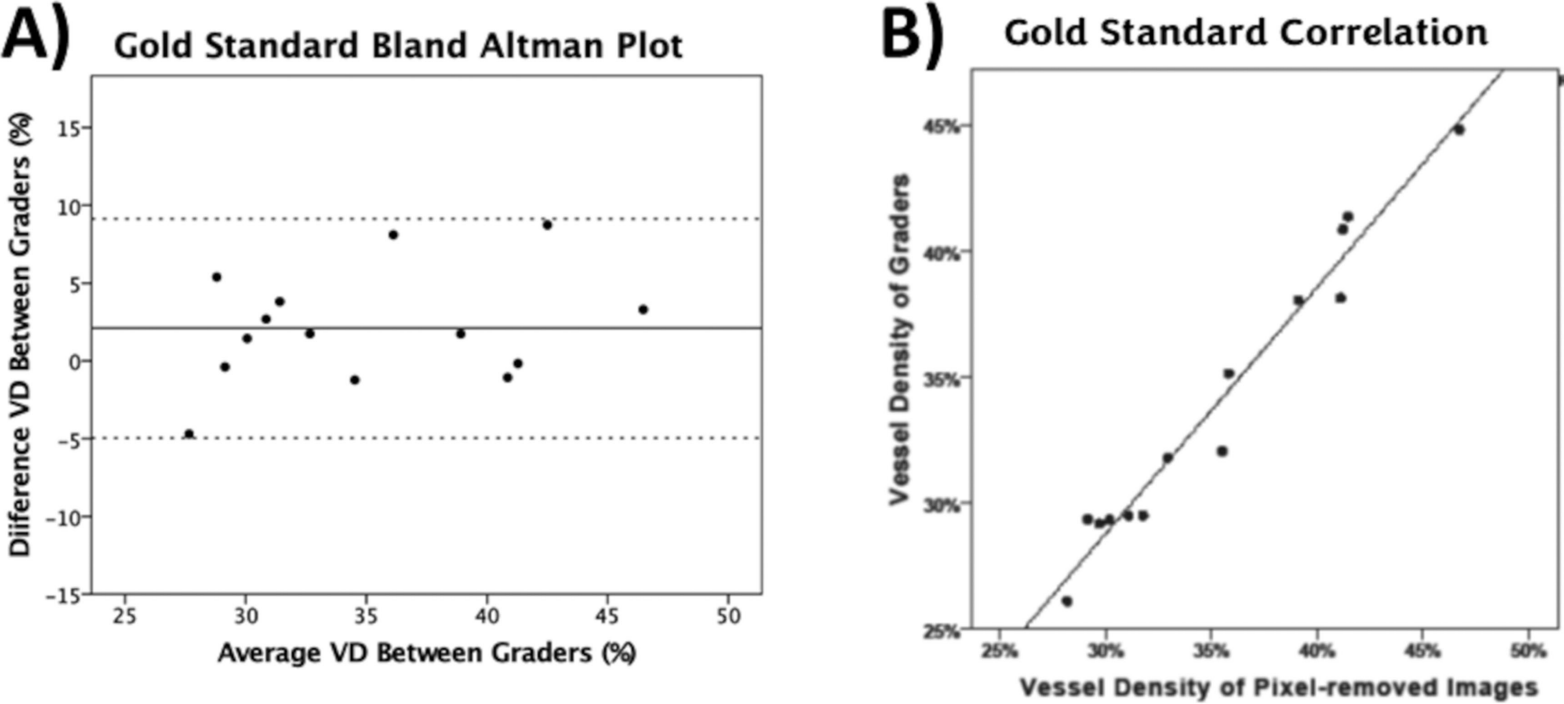
(A) Bland-Altman plots with the 95% upper and lower limits of the difference between the vessel density (VD) measurements between the graders. (B) The goodness-of-fit correlation between the VD measures of the three graders and the pixel-removed images for the gold-standard establishment.

A total of 14 images of 8 subjects were included and carefully selected to represent the variations of the conjunctival vessel location (i.e., nasal and temporal) and image characteristics, as shown in Table 1. The images that were meticulously assessed to remove the non-flow (background) pixels for the gold standard validation underwent binarization by 3 graders, their ICC was 0.816 [0.519–0.937] and the Bland-Altman plot showed a good agreement with less than ±10% variability between graders’ measurements and no significant bias (Fig. 4A). When comparing the new gold standard images created from the grader's threshold and the manually-removed pixel images, the VD agreement was 96.5% (*R*^2^ = 0.965, *P* < 0.001) with no significant bias (mean error <0.01 ± 0.96, *P* = 0.605), as shown in Figure 4B.

**Table 1.** General Overview and Pixel Specifications of the Images

	Subject	Eye	Location	Mean Gray Value	Gray Value SD	Median Gray Value	Gray Value Skewness	Gray Value Range
Image 1	A	Right	Nasal	72.9	49.9	66.0	0.6	0-255
Image 2	A	Right	Temporal	69.5	50.6	59.0	0.8	0-255
Image 3	B	Left	Nasal	53.3	49.9	43.0	0.9	0-255
Image 4	C	Right	Temporal	49.1	49.3	38.0	1.0	0-255
Image 5	D	Right	Nasal	52.1	63.8	27.0	1.3	0-255
Image 6	D	Right	Temporal	55.5	55.8	41.0	1.1	0-255
Image 7	D	Left	Nasal	53.0	64.4	27.0	1.3	0-255
Image 8	D	Left	Temporal	60.6	60.5	44.0	1.2	0-255
Image 9	E	Right	Nasal	66.9	50.5	57.0	0.8	0-255
Image 10	F	Left	Nasal	49.5	51.4	33.0	1.2	0-255
Image 11	G	Right	Nasal	42.0	45.3	28.0	1.2	0-255
Image 12	G	Left	Nasal	38.6	51.5	18.0	1.7	0-255
Image 13	G	Left	Temporal	41.3	46.8	26.0	1.0	0-255
Image 14	H	Right	Nasal	50.7	47.6	40.0	1.0	0-255

Standard deviation, SD.

The ICC values and their 95% confidence interval of the methodologies are summarized in Table 2. The repeatability of manual thresholding performed by 3 graders showed a poor agreement between them (ICC = 0.482 [0.032–0.798]) and a moderate agreement compared to the gold standard (ICC = 0.525 [−0.090 to 0.839]; Table 2). Overall, the local (adaptive) threshold methods showed better agreement compared to the global threshold and the mean threshold better agreement compared to Otsu threshold methods. Of interest, the mean and Phansalkar local threshold methods showed a good agreement and met the criteria for further accuracy assessment (ICC = 0.836 and ICC = 0.831, respectively). Conversely, the mean global threshold showed very poor agreement (ICC = 0.245).

**Table 2.** Reliability of the Threshold Methodologies

				95% CI	
Threshold Methodologies	Mean Gray Value VD (%)	±SD Gray Value VD (%)	ICC	Lower Bound	Upper Bound
1-Step Threshold Methods					
Manual Threshold	29.1	±4.9	0.525	−0.090	0.839
Mean Global Threshold	40.3	±2.7	0.245	−0.117	0.636
Otsu Global Threshold	29.7	±4.7	0.463	−0.056	0.791
Mean Local Threshold	34.3	±5.0	**0.836**	0.567	0.944
Otsu Local Threshold	28.8	±5.2	0.516	−0.099	0.836
Phansalkar Local Threshold	34.0	±6.1	**0.831**	0.547	0.943
2-Step Threshold Methods					
Close Morphology (1pxl) + Mean Local Threshold	34.1	±4.9	**0.838**	0.567	0.945
Close Morphology (1pxl) + Phansalkar Local Threshold	34.6	±6.2	**0.822**	0.541	0.939
Close Morphology (2pxl) + Mean Local Threshold	34.2	±4.8	**0.841**	0.574	0.946
Close Morphology (2pxl) + Phansalkar Local Threshold	36.3	±6.6	**0.763**	0.352	0.920
Bandpass Filter + Mean Local Threshold	34.7	±5.0	**0.835**	0.574	0.943
Bandpass Filter + Phansalkar Local Threshold	37.9	±6.0	**0.832**	0.556	0.943
Frangi Filter + Mean Local Threshold	49.1	±7.2	0.679	0.253	0.884
3-Step Threshold Methods					
Top hat Filter + Mean Local Threshold	29.7	±4.2	0.604	−0.086	0.882
Bandpass Filter + Gaussian Blur + Otsu Local Threshold	36.5	±4.9	**0.762**	0.213	0.927
Cornea Filter + Close Morphology + Mean Local Threshold	24.7	±5.3	0.316	−0.074	0.727
Gold Standard Images	33.9	±5.7	—	—	—

SD, standard deviation; pxl, pixel.

Methodologies with good reliability, ICC ≥0.750 (bold), that were selected for pxl location accuracy assessment.

The two-step methods that met the criteria for further accuracy assessment were the close morphology with 1-pixel radius and mean local threshold (ICC = 0.838; Fig. 2A_1_), 1-pixel radius and Phansalkar local threshold (ICC = 0.822; Fig. 2A_4_), close morphology with 2-pixel radius and mean local threshold (ICC = 0.841; Fig. 2B_1_), close morphology with 2-pixel radius and Phansalkar local threshold (ICC = 0.763; Fig. 2B_4_), Bandpass filter with mean local threshold (ICC = 0.835; Fig. 2D_1_), Bandpass filter with Phansalkar threshold (ICC = 0.832; Fig. 2D_3_), and Bandpass filter with Gaussian filter and Otsu local threshold (ICC = 0.762; Fig. 3A_2_). Conversely, the top hat filter with Otsu local threshold (Fig. 2C_2_) and the top hat filter, bandpass filter with Otsu local threshold (Fig. 3C_1_) showed the worst agreement (ICC = 0.136 and ICC = 0.142, respectively). The complete list of other methods that were tested but did not show good reliability are demonstrated as Supplemental Table S1, In general, the mean local threshold methods outperformed the Otsu and median local threshold methods. The latter methods, noticeably removed excessive amount of pixels overall.

**Figure 5.**
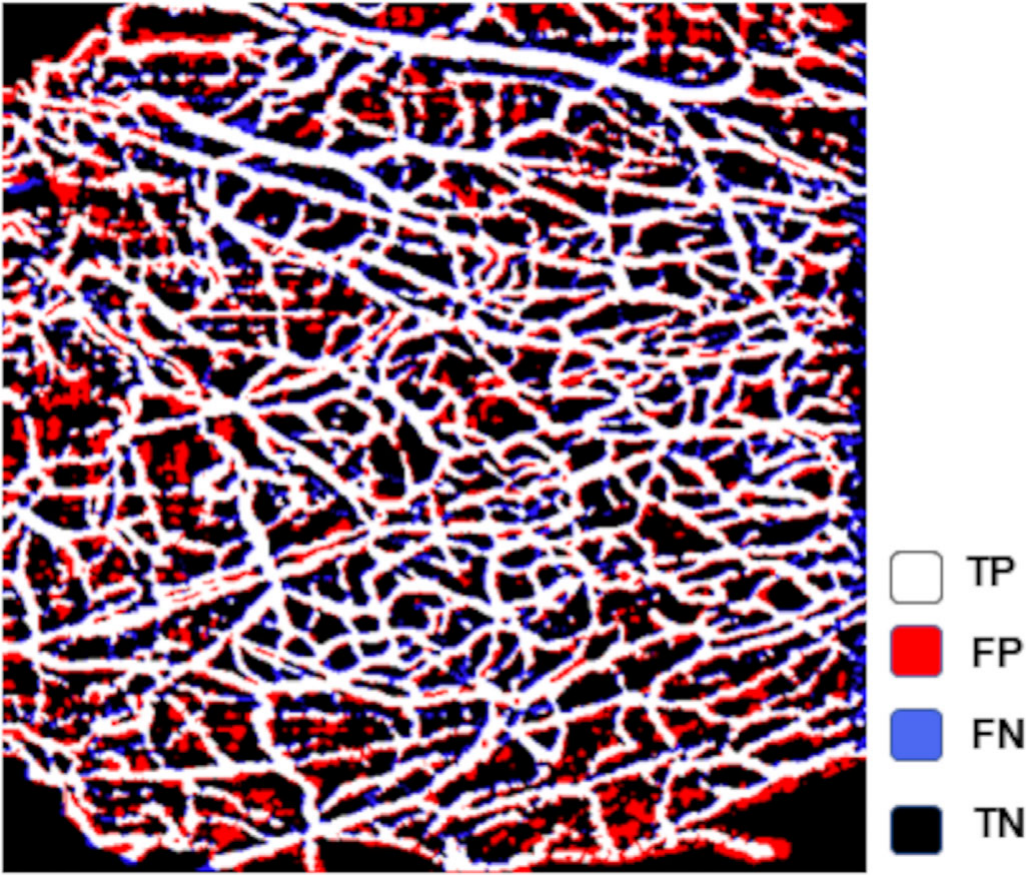
Representative OCTA image showing the overlapping pixels of the Bandpass filter + Phansalkar local threshold with the pixels from its gold-standard image. The *white pixels* represent true-positives (*TP*), the *red* false-positives (*FP*), the *blue* false-negatives (*FN*), and the *black* true-negatives (*TN*).

The performance details of the high reliability threshold methods to assess the pixel-location of the 14 sample images are shown in Table 3. The bandpass filter with Phansalkar local threshold method showed the highest accuracy in identifying the correct location of pixels that represented flow in the gold standard images (86.9% ± 6.8%, range 71.3%–94.1%), as shown in Figure 5. Interestingly, although the combined method using a 2-pixel radius close morphology with mean local threshold showed a higher ICC value for VD measurement compared to its 1-pixel radius counterpart (ICC = 0.841 and ICC = 0.838, respectively), the latter was more accurate in identifying the correct blood flow pixels; mean accuracy of 82.9% ± 7.5%, compared to 81.2% ± 7.5% from its 2-pixel counterpart.

**Table 3.** Performance of the High Reliability Threshold Methodologies

Threshold Methodologies	AUC	Sensitivity (%)	Specificity (%)	Accuracy (%)	Lower 95% CI	Upper 95% CI
1-Step Threshold Methods						
Mean Local Threshold	0.799	72.6	87.2	82.4	77.9	86.7
Phansalkar Local Threshold	0.808	73.5	88.1	82.9	78.7	87.3
2-Step Threshold Methods						
Close Morphology (1pxl) + Mean Local Threshold	0.804	72.8	88.0	82.7	78.4	87.0
Close Morphology (1pxl) + Phansalkar Local Threshold	0.728	57.9	87.8	73.8	68.8	78.8
Close Morphology (2pxl) + Mean Local Threshold	0.776	69.1	86.1	80.1	74.2	86.1
Close Morphology (2pxl) + Phansalkar Local Threshold	0.723	58.2	86.4	74.3	69.7	78.9
Bandpass Filter + Mean Local Threshold	0.809	74.2	87.6	83.5	79.2	87.8
Bandpass Filter + Phansalkar Local Threshold	0.826	79.0	86.1	86.9	83.0	91.0
3-Step Threshold Methods						
Bandpass Filter + Gaussian Blur + Otsu Local Threshold	0.723	64.6	80.0	76.5	70.7	82.4

Detailed performance of the pixel-location assessment of each methodology with averaged values from all images (e.g., AUC, sensitivity, specificity, accuracy, and 95% CI).

## Discussion

This study highlights that only select image threshold methods commonly used and published in the literature show a good agreement in measuring VD. Moreover, the combined threshold method using a bandpass filter with a Phansalkar local threshold showed the highest accuracy in identifying pixels representing blood vessels. As OCTA continues to grow in research and clinical trials, and more recently for anterior segment, accurate and reproducible vessel measurement quantification is of paramount importance. This study demonstrates how different image thresholding techniques can significantly alter the quantification of VD, which further highlights the need for a standardization.

There is a wide range of binarization methods in the literature and to date no consensus for image processing and vessel quantification for both posterior and anterior segment. The goal of this study was not to exhaust every available method, rather, to compare commonly reported methods and their combination using a common, widely available, and open-source software. As such, we undertook a stepwise approach to compare the different image processing techniques. Some studies utilize manual thresholding on anterior segment OCTA images.^14^^,^^41^^,^^42^ We showed that the agreement of manual thresholding was unsatisfactory for VD analysis, even though this approach was better than many automated threshold methods (global or local). Moreover, we showed agreement between graders to be poor with manual thresholding, highlighting the high variability of measures by using 3 graders. We also assessed the agreement between one-step global and local thresholds commonly used in the literature,^6^^,^^17^^,^^23^^,^^32^^,^^43^ and determined that local threshold outperformed global threshold methods. Accordingly, the local thresholding was used for consecutive combined thresholding methods, in accordance to previous reports.^6^^,^^32^ Overall, the local threshold generated a more homogenous image by accounting for the local gray value differences in each region. The most commonly used 1-step threshold method in the literature is Otsu (global and local).^15^^,^^17^^,^^23^^,^^43^ However, initial studies have shown a poor performance overall of this method, whereas recent approaches suggest Otsu threshold in combination with other image filters to improve its performance.^26^^,^^44^^,^^45^ We found a similar trend in our data, where the 3-step method with Otsu local threshold outperformed its 1-step method.

Next, commonly used combined methods were tested and relevant adaptations were assessed. The most common combined methods utilized in the literature are the Gaussian blur with Bandpass filter and Otsu local threshold (Fig. 3A_2_)^15^^,^^17^^,^^33^^,^^38^ and the top hat filter with a bandpass filter and an Otsu local threshold (Fig. 3C_1_).^23^^,^^43^^,^^46^ We found that the top hat filter with a bandpass filter followed by an Otsu local threshold showed very poor agreement (ICC = 0.142). Although Gaussian blur with Bandpass filter followed by an Otsu local threshold met the criteria for accuracy assessment (ICC = 0.762) it was significantly outperformed by bandpass filter with Phansalkar local threshold (AUC = 0.732 and AUC = 0.826, respectively).

A top hat filter has been frequently proposed as a method to reduce noise and remove small particles in the image without distortion.^32^^,^^43^ Although the combination of a top hat filter with a bandpass filter is the most commonly performed,^33^^,^^43^^,^^46^^,^^47^ we further explored the impact each filter on the VD agreement. In this study, an isolated top hat filter followed by a local threshold method showed worse agreement compared to the combined top hat and bandpass filters (Table 2). Conversely, the isolated bandpass filter followed by a local threshold method showed better agreement than the combined filters (top hat and bandpass), showing that the latter excessively removed pixels representing vessels. Of note, the combined method using the cornea gray value as a global suppression threshold, simulating the avascular signal-to-noise calculation from the foveal avascular zone in retina OCTA,^39^ showed a poor agreement. It is important to highlight that although this approach might be useful for corneal neovascularization thresholding,^16^ where the cornea presents a higher signal-to-noise ratio, we found it was not adequate for conjunctival vessel thresholding. A hessian-based Frangi vesselness filter is commonly used to identify vessel-like structures on a variety of images, including fundus photographs. However, the latter was designed to highlight vessels in noncontrasted images.^35^^,^^36^ The Frangi filter showed suboptimal reliability given the blood flow in OCTA has already high contrast, increasing the gray value cutoff of the image and thus thresholding many fine capillaries as background pixel.

Image binarization is an important step when quantifying VD in research studies and clinical trials using OCTA. Mehta and associates^30^ highlighted that different thresholding methods can significantly alter the absolute values as well as the directionality of trends, which could impact studies assessing quantitative vessel measurements. To date, there is no established parameter to compare and determine the quality of the thresholding methods. An ideal thresholding method is one that is widely available and accessible for research that can accurately binarize all pixels representing blood flow as foreground and all pixels that do not as background. Studies assessing the capability and sensitivity of OCTA in detecting true blood flow in vivo are lacking. One approach, widely accepted in the literature, is the manual removal of pixels on an OCTA image to produce a “gold standard” reference, then determining the parameters of a custom-built combined thresholding process (top hat filter with global threshold and local mean threshold) that more accurately reproduced the vessel measurements.^32^ Herein, although we took a similar approach to determine the “gold standard” for comparison, we used several images rather than a single image and performed the binarization on an open-source software widely used in research. Furthermore, we were able to determine the accuracy of each method in binarizing pixels correctly after selecting methods that met the criteria for good agreement. The combined bandpass filter followed by Phansalkar local threshold method showed the highest accuracy in the large subset of images (Fig. 5). Although not every thresholding method was tested in this study, it remains the largest study comparing a wide variety of thresholding methods on conjunctival OCTA images. However, the inherit underlying limitations from the manual adjustments to determine the gold-standard images, which currently lack validation, should be considered. Additionally, not all the available methods were tested. Nevertheless, this study is hopefully a step forward toward a more accurate and accessible thresholding method, highlighting the importance of establishing a standardized image binarization method to allow comparison between future studies and clinical trials.

Many studies within the published literature, interestingly, either forgo mention or do not clearly report the details of the thresholding method, resulting in difficulty in the interpretation and reproduction of the results. Also, while the use of the OCT device's built-in software is frequently used and advocated,^12^^,^^48^^,^^49^ the inherent variability between devices that can result from distinct algorithm processing steps of each platform cannot guarantee accuracy,^30^ especially in anterior segment where there are currently no specifically designed software. Another important point to highlight is that in our study, the cornea and eyelids were digitally removed from all OCTA images to focus on conjunctival vessels. This introduces determining conjunctival boundaries identification and manual selection, which is time consuming. Furthermore, not all individuals have the same interpalpebral exposure for conjunctival imaging, which may be a limitation of studies measuring conjunctival vessel parameters. Future trained algorithms designed to remove these features before image binarization are necessary for fully automated thresholding.

Studies have highlighted OCTA adapted for anterior segment capable of quantifying VD of small capillaries on the cornea and conjunctival better than slit-lamp photography and indocyanine green angiography.^46^^,^^50^ Conversely, another study demonstrated that OCTA was less precise in detecting small capillaries of corneal neovascularization compared to indocyanine green angiography.^51^ It is of note that these imaging techniques are not directly comparable when thresholding and quantifying vessels, given their differences in how flow is detected, the image contrast and resolution. Put differently, the same thresholding method for vessel quantification used in these images will not be optimal for all imaging techniques (i.e., OCTA, fluorescent angiography, color photography) and anatomical structures (i.e., macula, optic nerve, conjunctiva, cornea), as highlighted in our study. Accordingly, we believe that a consensus in OCTA thresholding and analysis for each anatomical structure is necessary for accurate vessel assessment.

There were several inherit limitations to our study. First, as previously discussed, is the lack of a gold standard. The manually created “gold standard” reference images as a means of comparison is suboptimal but necessarily commonly used, considering the available resources and knowledge from previous reports in the literature.^44^ However, by removing pixels that represented noise or did not resemble vessels on the OCTA image, in some images, a large number of pixels were excluded because of higher image noise. This further highlights the discrepancy of thresholding methods that generated low VD values (i.e., Otsu and manual threshold) and explains the high range of the 95% confidence intervals of ICC values; however, it simulates a more realistic assessment of conjunctival OCTA with variable noise. Furthermore, a relatively small number of images was analyzed, and not all threshold possibilities were tested in the current study. Manually removing pixels to create reference images is very time consuming and therefore challenging to create a large dataset of reference images for comparison. Nevertheless, our study power was 100%, assuming alpha of 0.05, for the gold standard validation. Finally, VD was the only vessel parameter studied herein. Although Reif et al.^32^ proposed the same combined threshold method for VD and fractal dimension, larger studies are warranted to address the role of thresholding for each vessel parameter.

Collectively, our results show the impact of image thresholding on VD and further highlight the need for a standardization in image thresholding, particularly in anterior segment OCTA because it is a novel field with distinct features that may affect binarization, such as higher signal-to-noise ratio and artifacts compared to better established OCTA imaging for posterior segment.

In conclusion, a combined thresholding method, particularly the bandpass filter with a local Phansalkar threshold, showed a good reliability and higher accuracy for pixel location and overall performance when compared to other combined methods or 1-step threshold methods. Many of the published methods in the literature did not show a satisfactory agreement and did not meet the criteria for accuracy assessment. Thus, the effects of image thresholding should be considered in vessel quantification and interpretation as it can impact future research studies and clinical trials using OCTA.

## Supplementary Material

Supplement 1
