# Supplementary material for: Automated Image Threshold Method Comparison for Conjunctival Vessel Quantification on Optical Coherence Tomography Angiography
Source: Transl Vis Sci Technol. 2022 Jul 20;11(7):15. doi: 10.1167/tvst.11.7.15 (PMC9315074; doi:10.1167/tvst.11.7.15)
Supplement: Supplement 1 [file tvst-11-7-15_s001.pdf]

**Supplemental Table** - Agreement of Other Threshold Methodologies

| Threshold Methodologies | Mean Grey Value VD (%) | ±SD Grey Value VD (%) | ICC | 95% Confidence Intervals | |
|---|---|---|---|---|---|
| | | | | Lower Bound | Upper Bound |
| 1. Median Local Threshold | 40.7 | ±4.9 | 0.453 | -0.085 | 0.823 |
| 2. Close Morphology (1pxl) + Median Local Threshold | 40.6 | ±4.8 | 0.450 | -0.087 | 0.821 |
| 3. Close Morphology (1pxl) + Otsu Local Threshold | 28.6 | ±5.0 | 0.494 | -0.106 | 0.826 |
| 4. Close Morphology (2pxl) + Median Local Threshold | 40.3 | ±4.7 | 0.468 | -0.091 | 0.829 |
| 5. Close Morphology (2pxl) + Otsu Local Threshold | 29.0 | ±4.9 | 0.509 | -0.097 | 0.832 |
| 6. Tophat Filter + Otsu Local Threshold | 19.9 | ±3.3 | 0.136 | -0.026 | 0.502 |
| 7. Bandpass Filter + Gaussian Blur + Mean Local Threshold | 38.0 | ±4.9 | 0.652 | -0.079 | 0.902 |
| 8. Bandpass Filter + Otsu Local Threshold | 29.1 | ±4.9 | 0.602 | -0.098 | 0.886 |
| 9. Frangi Filter + Otsu Local Threshol | 29.4 | ±4.3 | 0.360 | -0.189 | 0.737 |
| 10. Frangi Filter + Phansalkar Local Threshold | 39.1 | ±5.7 | 0.465 | -0.065 | 0.790 |
| 11. Bandpass Filter + Tophat Filter + Otsu Local Threshold | 20.4 | ±3.1 | 0.142 | -0.029 | 0.508 |
| 12. Bandpass Filter + Tophat Filter + Phansalkar Local Threshold | 29.3 | ±4.2 | 0.576 | -0.099 | 0.871 |
| **Gold Standard Images** | 33.9 | ±5.7 | - | - | - |

Intraclass correlation coefficient (ICC) agreement for other variations of threshold methodologies. VD= vessel density; SD= standard deviation; pxl= pixel.
